# Spinal Subdural Abscess following Transforaminal Lumbar Interbody Fusion

**DOI:** 10.1155/2020/7372821

**Published:** 2020-02-22

**Authors:** Isamu Miura, Motoo Kubota, Oji Momosaki, Kento Takebayashi, Takakazu Kawamata, Masahito Yuzurihara

**Affiliations:** ^1^Department of Spinal Surgery, Kameda Medical Center, 929 Higashi-cho, Kamogawa-shi, Chiba 296-8602, Japan; ^2^Department of Neurosurgery, Tokyo Women's Medical University, 8-1 Kawada-cho, Shinjuku-ku, Tokyo 162-8666, Japan

## Abstract

Spinal subdural abscesses are rare lesions. We report the case of surgical site infection complicated with meningitis and rapidly progressive spinal subdural abscess caused by *P. aeruginosa* following transforaminal lumbar interbody fusion (TLIF). A 72-year-old woman was admitted to our hospital complaining of drop foot syndrome and sciatica caused by stenosis of the L5/6 intervertebral foramen accompanied by L5 lumbar vertebral fracture. Accordingly, TLIF of L5-L6 and balloon kyphoplasty of L5 were performed. On the 3rd postoperative day (POD), she was diagnosed with surgical site infection complicated with bacterial meningitis. Subcutaneous fluid, blood, and cerebrospinal fluid cultures indicated *P. aeruginosa*. On the 7^th^ POD, a repeat MRI showed a large dorsal fluid collection consistent with a subdural infection and massive cauda equina compression. We performed debridement and instrument removal and found a dural laceration that was not observed during the first operation. An intraoperative insensible dural laceration may cause bacteria intrusion into the subdural space.

## 1. Introduction

Subdural abscesses are suppurative infections of the space between the dura and arachnoid. Spinal subdural abscesses are rare lesions [1]. Bacterial abscesses involving the spinal canal are associated with high morbidity and mortality [2]. The most common pathogen in spinal subdural abscesses is *Staphylococcus aureus*, and *Pseudomonas aeruginosa* is rarely detected in such lesions [3]. Herein, we report the case of surgical site infection complicated with meningitis and rapidly progressive spinal subdural abscess caused by *P. aeruginosa* following transforaminal lumbar interbody fusion (TLIF). Our findings showed that an intraoperative insensible dural laceration can cause bacteria intrusion into the subdural space.

## 2. Case Report

**Figure 1:**
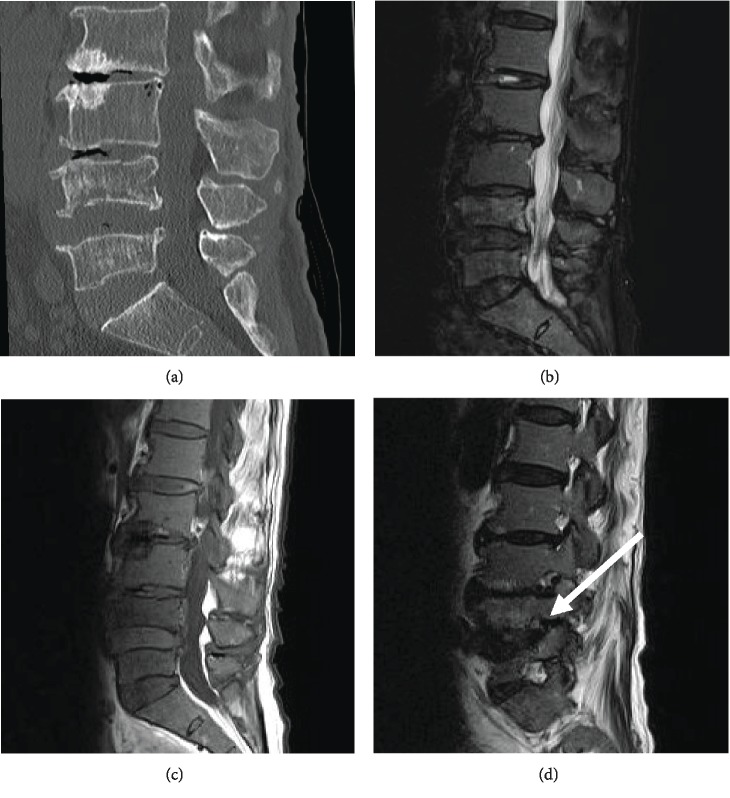
CT axial image demonstrating the decreased height of the L5 and L6 vertebral bodies (a). Sagittal magnetic resonance images showing iso- to low-intensity change in L5 vertebral body on a FLAIR (b), and low-intensity change on a T1-weighted image (c). T2 sagittal image showing compression of the left L5 nerve root at the L5/6 intervertebral foramen (d).

A 72-year-old woman with no significant medical history including diabetes started suffering from back pain and leg pain in the lower parts of her left leg at 16 weeks and 8 weeks prior to admission, respectively. She visited our hospital and was suspected to have an L5 vertebral fracture. She experienced left drop foot syndrome 1 week prior to admission and was admitted to our hospital for treatment. She had lumbago and left sciatica. Manual muscle testing of the tibialis anterior and extensor hallucis longus decreased to 1/5 each. The lumbar computed tomography showed a decreased height of L5 and L6 vertebral bodies (Figure 1(a)). Lumbar magnetic resonance images (MRI) of the L5 vertebral body showed slightly low intensity on T1-weighted images and slightly high intensity on the STIR images (Figures 1(b) and 1(c)). The left L5 nerve root was compressed within the L5/6 intervertebral foramen on the T2-weighted images (Figure 1(d)). Myelography was performed via the L2-L3 interlaminar space eight days before TLIF. The patient was considered to present left L5 radiculopathy caused by stenosis of the L5/6 intervertebral foramen accompanied by L5 lumbar vertebral fracture. Accordingly, TLIF of L5-L6 and balloon kyphoplasty of L5 were performed. Postoperatively, the patient had no neurological deterioration.

**Figure 2:**
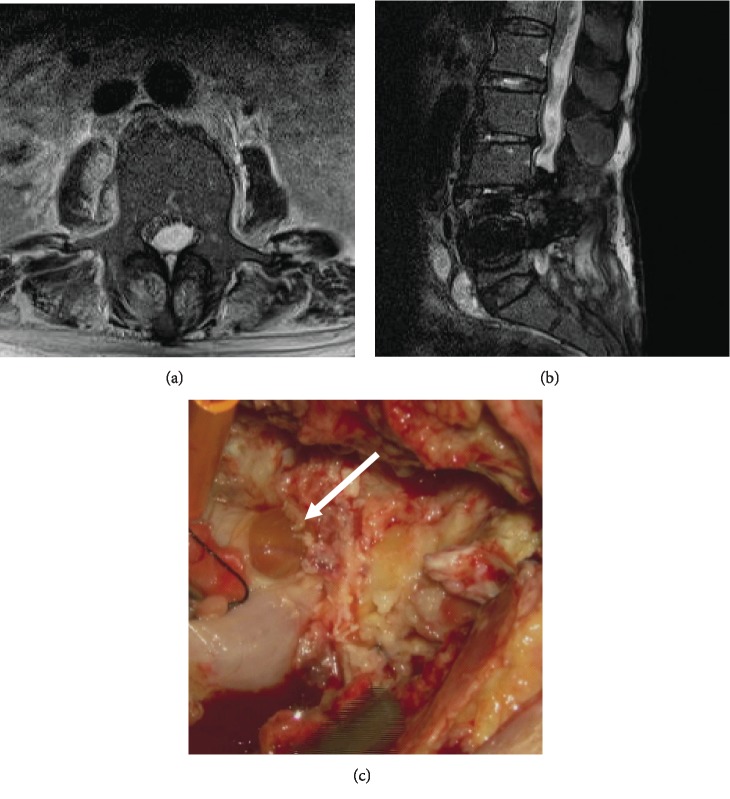
T2 axial image showing intradural high intensity mass compressing the cauda equina (a). T2 sagittal imaging showing high intensity areas of the anterior L5 vertebral, intradural space, and subcutaneous space (b). Operative view (c). Arrow showing arachnoid and laceration of dura at the edge of the residual L6 lamina.

**Figure 3:**
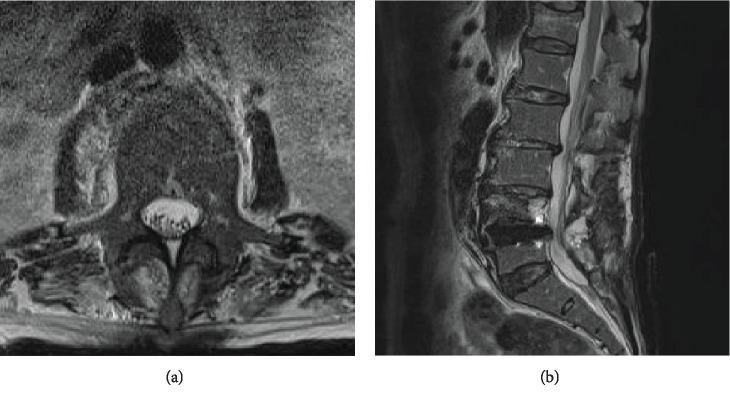
Postoperative MRI imaging after debridement. T2 axial image (a) and sagittal image (b) showing disappearance of mass compressing the cauda equina.

On the 3rd postoperative day (POD), she had a high fever of 39 degrees Celsius. She had neck stiffness and back pain with tenderness on pressure. Her white blood cell count and C-reactive protein level were over 60,000/mm^3^ and 23 mg/dL, respectively. Her cell count in cerebrospinal fluid (CSF) was over 5600/*μ*L (with 87% neutrophils). Lumbar MRI showed subcutaneous fluid without any findings of a subdual abscess or leakage of CSF. A gram stain of subcutaneous fluid showed gram-negative rods. Later, subcutaneous fluid cultures, CSF cultures, and blood cultures indicated *P. aeruginosa*. She was diagnosed with surgical site infection (SSI) complicated with bacterial meningitis. Cefepime and vancomycin were administrated. On the 7^th^ POD, a repeat MRI showed a large dorsal fluid collection consistent with a subdural infection and massive cauda equina compression (Figures 2(a) and 2(b)). Fluid on the anterior part of the L5 vertebral body and subcutaneous fluid had increased compared to the previous MRI finding. Debridement and instrument removal were performed under general anesthesia. All rods and pedicle screws were removed, but the titanium cages were left. A dural laceration on the edge of the residual L6 lamina was found (Figure 2(c)). The arachnoid was conserved, and there was no leakage of CSF. This laceration was not recognized during the first operation. The intravenous antibiotic therapy was continued and switched to an oral antibiotic six weeks after debridement. MRI showed no findings of subdural infection (Figure 3). Although we planned additional fixation surgery, fortunately, 8 months later, the patient had no neurological deterioration following conservative treatment using a lumbar corset and administration of romosozumab to prevent new compression fractures and promote bone fusion. We need further follow-up because of the possibility of a slip or subsidence. If the slip progresses, we will consider surgical intervention using percutaneous pedicle screws with a different trajectory.

## 3. Discussion

*P. aeruginosa* is an important pathogen of SSI [4]. Hey et al. reported that the prevalence rate of *P. aeruginosa* in SSI was 35%. On the other hand, most pathogens involved methicillin-susceptible *S. aureus* and methicillin-resistant *S. aureus* in spinal subdural abscess [1, 2, 5]. Velissaris et al. reviewed 65 cases of spinal subdural abscess and found only one case of spinal lumber abscess due to *P. aeruginosa* [2]. Therefore, our case is thought to be rare.

In our case, a dural laceration that was not observed during the first operation was found during wound debridement and instrument removal. Wu et al. reported a case of spinal subdural empyema after a dural tear during thoracic laminectomy [6]. They considered that the anatomical barrier of the dura was disrupted during the initial surgery via a dural tear, facilitating subdural extension of the infection. In our case, bacterial meningitis preceded the appearance of subdural abscess on the MRI. Wu et al. reported the case of spinal subdural abscess following meningitis caused by *S. aureus* [6]. In our patient, myelography was performed via the L2-L3 interlaminar space eight days before TLIF. The patient had no fever and no headache after myelography, and the puncture level was different from the operation level. Although the possibility of infection by the puncture for myelography cannot be completely excluded, it is thought that, in this instance, dural laceration during TLIF allowed bacteria to intrude into the subdural space. A spinal CSF fistula may develop into bacterial meningitis although we could not find CSF leakage. This indicates that there may be an unnoticed dural tear or laceration in cases of postoperative spinal subdural abscesses.

Subdural abscess is a serious condition, and Bartels et al. reported a mortality rate of 25% [7]. Fortunately, we could treat the patient in our case. Early diagnosis and emergent treatment are vital to prevent the formation and progression of neurologic deficits and death [2].

This case involves left L5 foraminal stenosis after an L5 compression fracture. The most widely used surgical approach for osteoporotic spinal fractures is fusion surgery including vertebroplasty and kyphoplasty [8]. For foraminal stenosis, decompression surgery is commonly used via facetectomy and elevation of the disc height by interbody fusion [9]. Sasaki et al. reported a case of vertebroplasty and posterior interbody fusion (PLIF) for radiculopathy caused by osteoporotic vertebral fractures [10]. They reported that the surgical outcome of PLIF is better than that of other surgical methods without fixation because spinal stabilization is preserved. We performed TLIF. However, the surgical site infection rate in instrumentation surgery is higher. Recently, a report has described foraminal decompression via a lateral approach using spinal endoscopy [11]. In this case, minimally invasive endoscopic surgery may be better.

*P. aeruginosa* can cause a rapidly progressive spinal subdural abscess when a dural laceration occurs during spinal surgery because bacteria can directly intrude into the subdural space.

## References

[1] Ramos A. D., Rolston J. D., Gauger G. E., Larson P. S. (2016). Spinal subdural abscess following laminectomy for symptomatic stenosis: a report of 2 cases and review of the literature. *American Journal of Case Reports*.

[2] Velissaris D., Aretha D., Fligou F., Filos K. S. (2009). Spinal subdural Staphylococcus aureus abscess: case report and review of the literature. *World Journal of Emergency Surgery*.

[3] Coumans J. V., Walcott B. P. (2011). Rapidly progressive lumbar subdural empyema following acromial bursal injection. *Journal of Clinical Neuroscience*.

[4] Hey H. W., Thiam D. W., Koh Z. S. (2017). Is intraoperative local vancomycin powder the answer to surgical site infections in spine surgery?. *Spine*.

[5] Kraeutler M. J., Bozzay J. D., Walker M. P., John K. (2015). Spinal subdural abscess following epidural steroid injection. *Journal of Neurosurgery Spine*.

[6] Wu A. S., Griebel R. W., Meguro K., Fourney D. R. (2004). Spinal subdural empyema after a dural tear. Case report. *Neurosurgical Focus*.

[7] Bartels R. H., de Jong T. R., Grotenhuis J. A. (1992). Spinal subdural abscess. Case report. *Journal of Neurosurgery*.

[8] Shen M., Kim Y. (2007). Osteoporotic vertebral compression fractures: a review of current surgical management techniques. *American Journal of Orthopedics*.

[9] Fujibayashi S., Neo M., Takemoto M., Ota M., Nakamura T. (2010). Paraspinal-approach transforaminal lumbar interbody fusion for the treatment of lumbar foraminal stenosis. *Journal of Neurosurgery Spine*.

[10] Sasaki M., Aoki M., Nishioka K., Yoshimine T. (2011). Radiculopathy caused by osteoporotic vertebral fractures in the lumbar spine. *Neurologia Medico-Chirurgica*.

[11] Ishimoto Y., Yamada H., Curtis E. (2018). Spinal endoscopy for delayed-onset lumbar radiculopathy resulting from foraminal stenosis after osteoporotic vertebral fracture: a case report of a new surgical strategy. *Case Reports in Orthopedics*.

